# The Elusive Nature of ABCC8-related Maturity-Onset Diabetes of the Young (ABCC8-MODY). A Review of the Literature and Case Discussion

**DOI:** 10.1007/s11892-024-01547-1

**Published:** 2024-07-09

**Authors:** Marella Marassi, Mario Luca Morieri, Viola Sanga, Giulio Ceolotto, Angelo Avogaro, Gian Paolo Fadini

**Affiliations:** 1 Department of Medicine, University of Padova, Via Giustiniani 2, Padua, 35100 Italy; 2 Veneto Institute of Molecular Medicine, Padua, 35100 Italy

**Keywords:** Genetics, Type 2 diabetes, Variants, Youth

## Abstract

**Purpose of Review:**

Maturity-onset diabetes of the young (MODY) are monogenic forms of diabetes resulting from genetic defects, usually transmitted in an autosomal dominant fashion, leading to β-cell dysfunction. Due to the lack of homogeneous clinical features and univocal diagnostic criteria, MODY is often misdiagnosed as type 1 or type 2 diabetes, hence its diagnosis relies mostly on genetic testing. Fourteen subtypes of MODY have been described to date. Here, we review ABCC8-MODY pathophysiology, genetic and clinical features, and current therapeutic options.

**Recent Findings:**

ABCC8-MODY is caused by mutations in the adenosine triphosphate (ATP)-binding cassette transporter subfamily C member 8 (*ABCC8*) gene, involved in the regulation of insulin secretion. The complexity of ABCC8-MODY genetic picture is mirrored by a variety of clinical manifestations, encompassing a wide spectrum of disease severity. Such inconsistency of genotype-phenotype correlation has not been fully understood. A correct diagnosis is crucial for the choice of adequate treatment and outcome improvement. By targeting the defective gene product, sulfonylureas are the preferred medications in ABCC8-MODY, although efficacy vary substantially. We illustrate three case reports in whom a diagnosis of ABCC8-MODY was suspected after the identification of novel *ABCC8* variants that turned out to be of unknown significance. We discuss that careful interpretation of genetic testing is needed even on the background of a suggestive clinical context.

**Summary:**

We highlight the need for further research to unravel ABCC8-MODY disease mechanisms, as well as to clarify the pathogenicity of identified *ABCC8* variants and their influence on clinical presentation and response to therapy.

## Introduction

Maturity-Onset Diabetes of the Young (MODY) is an heterogenous group of monogenic forms of diabetes mellitus caused by genetic defects mainly leading to the secretory dysfunction of pancreatic β-cells [1]. MODY is a rare condition, accounting for 1–5% of all cases of diabetes, and easily misdiagnosed as type 1 (T1DM) or type 2 (T2DM) diabetes mellitus in clinical practice [2]. According to diagnostic criteria, MODY is characterized by: (i) onset before 25 years of age in at least one of family members, (ii) β-cell dysfunction without evidence of autoimmunity, (iii) autosomal dominant hereditary diabetes in at least two generations, and (iv) sustained endogenous insulin secretion reserve [1]. Given the great proportion of MODY individuals confirmed by gene sequencing who does not fulfill the traditional diagnostic criteria, genetic testing remains the gold standard for diagnosis [3]. To aid clinicians in identifying which individuals should be tested, predictions models based on weighted clinical and laboratory criteria have been validated to determine an individual’s pre-test probability of having MODY compared with the more common T1DM and T2DM. These are useful tools to improve the rational selection for molecular genetic testing for MODY [4].

To date, 14 subtypes of MODY have been reported in the literature, with heterogeneity in their prevalence, clinical features and treatment requirements and caused by diverse pathogenic genetic defects [2, 5]. However, based on a recent revision of gene-disease associations, the list of MODY-causative genes should be restricted to the following: *HNF4A, GCK, HNF1A, PDX1, HNF1B, NEUROD1, CEL, INS, ABCC8, KCNJ11*, along with *RFX6*, recently proposed as an additional MODY gene [6].

Despite substantial population-based differences, GCK-MODY and HNF1A-MODY are the most prevalent, accounting for 90% of all MODY cases [7]. Conversely, the occurrence of the ABCC8-MODY (sometimes referred to as MODY12 [6]) subtype is rare, accounting for 1% of the entire MODY population [8]. ABCC8-MODY is caused by pathogenic mutations involving the adenosine triphosphate (ATP)-binding cassette transporter subfamily C member 8 (*ABCC8*) gene [9]. In this article, we conducted a literature review for reported ABCC8-MODY cases, describing their genetic and clinical features, as well as treatment options. Furthermore, we report new cases in whom the detection of novel *ABCC*8 variants in a suggestive clinical history led to a suspect of ABCC8-MODY.

### ABCC8 Mutation-associated Disease Spectrum

The *ABCC8* gene, located on the short arm of chromosome 11, consists of 39 exons encoding for 1582 amino acids [10]. It encodes sulfonylurea receptor 1 (SUR1) [11], a regulatory subunit of the hetero-octomeric ATP-sensitive potassium (K_ATP_) channel in pancreatic β-cells [12]. The SUR1 protein is predicted to contain three transmembrane domains (TMD) named TMD0, TMD1, and TMD2, with a cytosolic linker (L0), and two nucleotide binding domains (NBD) named NBD1, NBD2 [11]. While TMD0 and L0 are important for interactions with the pore-forming subunits (Kir6.2) of the K_ATP_ channel [11], NBDs are crucial for the binding of nucleotides, which modulate channel opening [12]. Since insulin release is mediated by the ATP-dependent closure of this K channel, *ABCC8* mutations altering SUR1 function result in various abnormalities in insulin secretion [9]. The spectrum of *ABCC8* mutations is wide, which is mirrored by complex and heterogenous phenotypic manifestations.

Loss-of-function (LOF) mutations in *ABCC8* can result either in the absence of the K_ATP_ channel in the β-cell membrane or in its defective opening in response to Mg-nucleotide binding [12]. These defects cause neonatal hyperinsulinisms, characterized by unregulated insulin secretion from β-cells, leading to severe hypoglycemia [13]. Some dominantly inactivating variants can cause hyperinsulinism with predisposition to progressive insulin deficiency and leading to diabetes later in life [14].

Conversely, both dominant and recessive gain-of-function *ABCC8* variants have been associated with permanent neonatal diabetes (PNDM) and transient neonatal diabetes (TNDM) [15, 16]. TNDM can relapse later in life after transient remission [15]. Finally, activating *ABCC8* mutations have been shown to cause ABCC8-MODY, characterized by variable clinical phenotypes of diabetes with an onset in childhood/early adulthood [17]. However, predicting a phenotype based solely on the functional effect of the *ABCC8* variant can be misleading, since there are reports of diabetes owing to LOF mutations in *ABCC8* in the absence of the hyperinsulinism-remission-diabetes sequence [18, 19].

Table 1 shows identified *ABCC8* variants and associated clinical phenotypes reported among key studies [11, 13–16, 18–57]. What emerges from the available data is that not only *ABCC8*-related diabetes can result from both activating and inactivating mutations, but variants located in the same protein domain – or even in the same mutation site – can give different phenotypes throughout the disease spectrum. A comprehensive explanation for genotype-phenotype heterogeneity is still lacking. Chronic β-cell hyperfunction turning into progressive exhaustion may be a putative mechanism for the hypoglycemia-diabetes duality observed with certain *ABCC8* variants. In addition, carriers of the same genotype can show variability in phenotype depending on the disease phase, defect penetrance, and influence of modulating factors [57]. Further investigation is needed to shed light on the mechanisms underlying the heterogeneity in clinical manifestations owing to *ABCC8* mutations.

**Table 1:** *ABCC8* variants and respective clinical phenotypes throughout *ABCC8*-related disease spectrum identified among key studies

DOMAINS	PNDM/TNDM	ABCC8-MODY	HH-DIABETES SEQUENCE	HH
TMD0	p.S8R, p.P45L, p.N72S p.V86A/G, p.A90V, p.F132L/V; p.L135P	p.E100K	p.L171F	p.C6*, p.G7R, p.V21D, p.N24K, p.F27S, p.R74W/Q, p.E128K, p.L171P, p.V187D;
L0	p.P207S, p.E208K, p.D209E, p.D212I/N, p.L213R, p.R216C, p.Q221K, p.L225P, p.T229I/N, p.Y263D, p.R285Q, p.G296R, p.R306H	p.R298C; p.R306C; p.P201S		p.E208*, p.Q219*p.W231R, p.Y232*p.R248*, p.C267*, p.Q219*, p.R298C, p.D310N; p.R306C + p.G1478R
TMD1	p.V324M, p.E350D, *p.A355T*, p.E382K, p.I395F, p.H410Y, p.C435R, p.L438F, p.L451P, p.S459R, p.Q485H, p.F536L, p.F577L, p.L582V, p.I585T, p.V587G	p.Y356C, p.C418R, p.C435R, p.Y475H, p.A478T; p.Q485R, **p.T548P**, p.L582V; p.R519C	p.R370S	*p.A355T*, p.D370G; p.I462V, p.Q474R, p.A478D, p.E490V, p.R495Q, p.E501K, p.L503P, p.L508P; p.L511M, p.R521Q, p.R526C, p.R620C, p.F686S, p.G716V;
NBD1	p.V607M, p.R653Q, p.E747*, p.R826W, p.G832C/D, p.H862Y, p.R877Q, p.D897V, p.E939K	p.N718S, p.R825W; p.G833S, p.Q834K, p.E971V; p.S962L	p.R934*	p.V715A/G, p.G716D, p.G718F, p.T888P; p.E825K, p.E825*, p.R837*, p.K890T, p.L891P, p.Q917*, p.R934*
TMD2	p.H1024Y, p.S1053N, p.F1163L, p.F1067I, p.N1122D, p.F1176L, p.Q1178R, p.F1181S, p.R1183W/Q, p.A1185E, p.P1198L, p.G1255S, p.M1290V	p.G1009S, p.K1023Q, p.S1053W, p.F1068I, **p.I1075T**, p.F1176C, p.R1183W, p.E1206K; p.1221 W; **p.E1228K**, p.I1236V, p.N1245D	p.L1148R, p.R1251*	p.R1215W/Q, p.A1263T
NBD2	p.R1314H, p.E1327K, p.R1380C/H, p.G1401R, p.I1425V, p.E1506Q, p.V1523A/L, p.A1536P	p.E1326K, *p.R1353H*/K, p.K1358R, p.R1379H, p.G1383A, p.S1386F; p.A1390V, p.I1404V; p.I1423V, p.G1433S, p.*A1457T*, p.A1473T, *p.G1478R;* p.E1507Q, p.M1514T, *p.T1515M*; p.V1523L, p.A1536T	p.A1367D, p.R1385Q, p.R1419H, p. G1479R p.E1506K; p.A1508P p.R168C + p.R1421C	p.K1337N, p.K1347R, p.L1350Q, *p.R1353H/P*, p.K1374R, p.G1382S, p.G1384R/E, p.DELS1387, p.S1387F/Y/P, p.S1389Y, p.L1390P/R, p.G1400R, p.P1413L, p.L1431F, *p.A1458T*, p.Q1459H/E, p.V1464*, p.D1472H, p.G1478V, *p.G1479R*/E, p.N1481I, p.G1485E, p.R1493W, p.D1505E, p.E1506K, p.I1512T/S, p.M1514K, *p.T1516M*, p.E1517K p.T1531A, p.A1537V, p.R1539Q

Identical *ABBC8* variants* reported to be associated with different clinical phenotypes are shown in Italics. The three new *ABCC8* variants discussed in the paper are reported in bold.

* The alternative splicing of exon 17 produces two different ABCC8 transcripts differing in length by a single amino acid. Thus, the nomenclature of ABCC8 variants in exons 17–39 may differ across the studies depending on which isoform has been used.

**Table 2:** Pathogenic (P)/likely pathogenic (LP) ABCC8 variants reported in ABCC8-MODY and T2DM cases

Topological domain	*P*/LP ABCC8 variants associated with ABCC8-MODY	References	*P*/LP ABCC8 variants reported in T2DM*	References
TMD0	p.E100K;	[9]	p.R16P, p.R74Q, p.L106M, p.M115V, p.H125Q	[58]
L0	p.R298C; p.R306C, p.P201S	[17, 42]	p.V222M, p.A240V, p.A269D, p.R298C, p.D310N, p.F221L	[58]
TMD1	p.Y356C, p.C418R, p.C435R, p.Y475H, p.A478T; p.Q485R, p.L582V; p.R519C	[9, 17, 44, 48, 49, 56]	p.G316R, p.T479I, p.R521Q	[58]
NBD1	p.N718S, p.R825W; p.G833S, p.Q834K, p.E971V; p.S962L	[17, 45, 48, 59, 60]	p.K889T, p.R825L, p.R825Q, p.W688S, p.R653Q	[58]
TMD2	p.G1009S, p.K1023Q, p.S1053W, p.F1068I, p.F1176C, p.R1183W, p.E1206K; p.1221 W; p.I1236V, p.N1245D	[9, 42, 50, 59] [48, 55, 60, 61]	p.D1030N, p.R1182Q, p.V1249D	[58]
NBD2	p.E1326K, p.R1353H/K, p.K1358R, p.R1379H, p.G1383A, p.S1386F; p.A1390V, p.I1404V; p.I1423V, p.G1433S, p.A1457T, p.A1473T, p.G1478R; p.E1507Q, p.M1514T, p.T1515M; p.V1523L, p.A1536T	[5, 8, 9, 17, 19, 42, 43, 46, 47, 50–54, 59–61] [47]	p.R1393H, p.G1375A, p.R1313H, p.R1352H, p.V1249D, p.G1433R, p.A1492G, p.A1536V	[58]

* Data are extracted from the participants with T2DM from the RaDiO study, the UK Biobank and the Healthy Nevada Project Study

**Table 3:** Summary of the new case series

Characteristic	Case 1	Case 2	Case 3
ABCC8 mutation	c.3224T > C	c.1642 A > C	c.3682G > A
Aminoacid substitution	p.Ile1075Thr	p.Thr548Pro	p. Glu1228Lys
Protein region	transmembrane portion of TMD2	trasmembrane protein portion of TMD1	transmembrane portion of TMD2
Sex	Male	Male	Female
Age at onset	53	18	34
Family history	Yes	No	No
Mutation in the family	Yes	No	Not tested
Antibodies	Negative	Negative	Negative
Diabetes duration	9	20	1
BMI, kg/m^2^	22.2	21.7	19.0
Hypertension	No	No	No
Dyslipidemia	No	No	No
Cardiovascular disease	No	No	No
Complications (years of follow-up)	No (12)	No (17)	No (1)
Pharmacological therapy	sitagliptin / metformin	sitagliptin / acarbose	None
Trial with sulfonylurea	Not needed	Yes, interrupted due to poor glycemic response	Not needed
Last HbA1c, %	6.3	6.5	5.1

We systematically conducted a literature search for original articles and review articles describing ABCC8-MODY cases and their respective *ABCC8* mutations with publication dates up to July 2023. We run the search on PubMed and specific search terms were: “Maturity-Onset Diabetes of the Young (MODY)”, “ABCC8-MODY”, “MODY12”, “*ABCC8* variants”. Language restriction (English) was applied. The articles’ cross- references were also included. The genetic information of *ABCC8* gene was as follows: accession number: NM_000352.6, NP_00343.2.

**Fig. 1:**
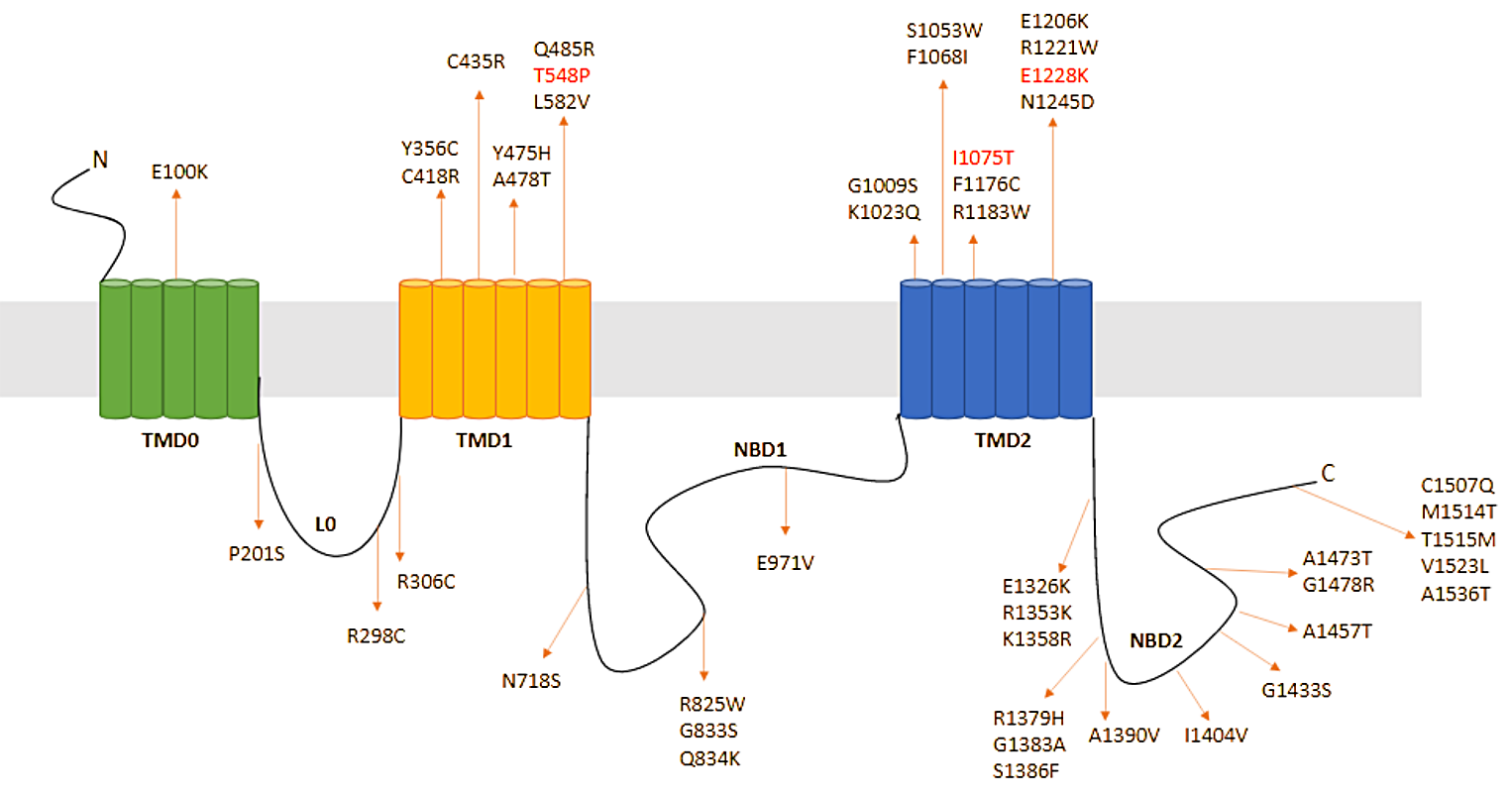
A schematic representation of the transmembrane topology of SUR1 showing the location of the *ABCC8* variants associated with ABCC8-MODY\. The three transmembrane domains (TMD) are indicated by TMD0, TMD1 and TMD2. The nucleotide-binding domains (NBD) include NBD1 and NBD2, and the cytosolic linker L0 is between TMD0 and TMD1. The three new *ABCC8* variants described in the paper are highlighted in red. SUR1, sulfonylurea receptor 1; *ABCC8*, adenosine triphosphate (ATP)-binding cassette transporter subfamily C member 8

Figure 1 illustrates the mutational hotspots throughout the *ABCC8* gene which have so far been associated with ABCC8-MODY.

### ABCC8-MODY: A Review of the Literature

ABCC8-MODY has a low prevalence among MODY subtypes, accounting for 1% of the entire MODY, even though its recognition has increased with the growing application of next-generation sequencing technology [59]. According to the literature, the prevalence of MODY owing to *ABCC8* mutations ranges from 0.9 to 3.3% among clinically suspected MODY cohorts [53, 60], possibly equal to 30–50 cases / 1 million individuals.

There are limited large-scale studies on ABCC8-MODY, while the majority of studies are case reports. Up to date, more than 55 variants of *ABCC8* have been associated with ABCC8-MODY, most of which have been reported only once [8]. On the other hand, variants of known MODY-causative genes (including *ABCC8*) have been reported among people diagnosed with traditional T2DM [58]. Given the lack of distinctive clinical features of ABCC8-MODY, distinguishing *ABCC8* disease-causing variants from benign variants can be challenging. This is particularly true for novel mutations, or when the family pedigree is not available to check co-segregation.

Recently, a study from China identified 17 rare *ABCC8* variants among 543 individuals with early-onset-diabetes, of which only 8 variants were deemed to be pathogenic and cause ABCC8-MODY [42]. Similarly, among a cohort of individuals with T2DM well controlled with sulfonylurea, 4 probands carried *ABCC8* mutations, of which 2 variants were likely causal, the others putatively representing rare benign variants [17]. Thus, the interpretation of sequence variants is one major issue in the differential diagnosis between monogenic and more common diabetes subtypes. Whether the identification of variants of uncertain significance in MODY-associated genes should be considered as the cause of monogenic diabetes or as a risk factor for the occurrence of T2DM is still under debate [62]. Table 2 summarizes pathogenic (P)/likely pathogenic (LP) *ABCC8* variants identified among ABCC8-MODY and T2DM cases in previous studies.

As mentioned above, a distinctive feature of ABCC8-MODY is its heterogeneity in disease presentation. The clinical picture associated with ABCC8-MODY is diverse even among relatives carrying the same mutation [8, 57]. Thus, the type and location of *ABCC8* mutations cannot fully explain per se the variability in phenotypes. Genetic modifiers and environmental influences might play a role in determining the observed inconsistency of genotype-phenotype correlation [17].

According to a recent systematic review of ABCC8-MODY cases, disease severity can range from mild glucose impairment to insulin-dependent diabetes [8]. Although there is evidence that individuals with ABCC8-MODY are prone to develop microvascular complications, the overall incidence and prognosis of diabetic complications remain uncertain due to a lack of systematic reports.

According to the literature, renal impairment and retinopathy frequently affect individuals with ABCC8-MODY [8, 40, 42, 59], their prevalence reaching 30% and 50%, respectively [59]. Given that SUR1 is expressed in retinal vessels, it has been hypothesized that *ABCC8* mutations may play a direct role in the development of ocular complications [59]. On the other hand, in light of the low *ABCC8* expression levels in the kidney [63, 64], a direct role of *ABCC8* in the pathogenesis of renal impairment is difficult to postulate. According to mouse models, *ABCC8* variants may induce the expression of proinflammatory genes in the kidney via hyperglycemia-mediated epigenetic changes, thereby indirectly predisposing to diabetic kidney disease [65]. Nevertheless, further studies are required to elucidate the precise role of *ABCC8* variants in the development of renal complications.

Although there is evidence that K_ATP_ channels are expressed in the nervous system and *ABCC8*-neonatal diabetes has been extensively associated with neurological impairment [41], it is still controversial if neurological complications are also a feature of ABCC8-MODY. Indeed, despite a single case report of an individual with ABCC8-MODY and epilepsy [5], numerous studies have reported the absence of neurological defects among different ABCC8-MODY cohorts [40, 42]. Further research is warranted to clarify if there is an association between ABCC8-MODY and neuropathy.

It is broadly known that MODY is underrecognized in clinical practice and often misclassified as T1DM or T2DM. Since ABCC8-MODY can present initially with severe hyperglycemia with classical symptoms of diabetes, it is often inappropriately treated with an insulin-based regimen when undiagnosed [59], typically with poor glucose control [43, 66]. The correct identification of ABCC8-MODY is crucial for the choice of adequate treatment: as sulfonylureas specifically bind to the SUR1 subunit and close the K_ATP_ channel to release insulin [67], they are the drug of choice in ABCC8-MODY, with proven efficacy [17, 44, 59].

Switching from insulin to sulfonylurea-based therapy after genetic diagnosis of ABCC8-MODY has been associated with improved C-peptide levels and metabolic control, and lower rates of hypoglycemia [8, 45]. At the same time, insulin discontinuation in these individuals favors body weight loss [46] and reduces glucose variability [5, 59], which is a potential driver for diabetic vascular complications [68].

However, it is worth noticing that the efficacy of sulfonylureas may vary according to the type of *ABCC8* mutation. Indeed, in some reports, most of, but not all, individuals with *ABCC8*-induced diabetes successfully switched from insulin to sulfonylureas [40, 69]. On the other hand, there is a growing body of evidence suggesting that incretins may be beneficial in ABCC8-MODY, offering a promising alternative to sulfonylureas [47, 48]. In dysfunctional β-cells due to *ABCC8* mutations, the incretin effect is highly preserved and the activation of the downstream signaling pathways enhances insulin secretion, potentially overcoming the stimulatory effect of GOF-ABCC8 variants on the K_ATP_ channel pore [47].

Additionally, it should be mentioned that incretin-based therapy – and not sulfonylureas – has been shown to be an effective approach in individuals with diabetes owing to *ABCC8* inactivating variants [20, 21]. The experimental evidence that β-cells with inactivating *ABCC8* mutations retain adequate basal insulin secretion but are characterized by impaired glucose-stimulated insulin release [70], may provide a rationale for the use of incretin-based drugs in this population. Thus, the type of functional and structural disorder resulting from diverse *ABCC8* variants may influence the response to therapy, suggesting that the genetic features are important when choosing the appropriate treatment.

Recently, a report of a ABCC8-MODY family from China demonstrated that optimal glycemic control can be achieved with a metformin-based therapy combined with exercise and diet [46]. Furthermore, Ovsyannikova et al. reported a case of an individual with ABCC8-MODY in whom combining sodium-glucose co-transporter 2 inhibitors (SGLT2i) with a sulfonylurea resulted in improved glycemic variability parameters without hypoglycemia [5]. Even though data on the use of this class of medications in MODY are scarce, the available findings suggest that SGLT2i may be a promising treatment option for this disease, with the potential to reduce glucose fluctuations and thus, the burden of microvascular complications.

Therefore, the correct recognition of a MODY subtype and the identification of pathogenic mutations through genetic analysis is of great significance for therapeutic decisions, as it can provide the basis for a personalized adequate treatment. The choice of a tailored therapy will maximize the efficacy of pharmacological treatments and limit the incidence of complications, improving prognosis.

## Case Discussion

### Case #1

**Fig. 2:**
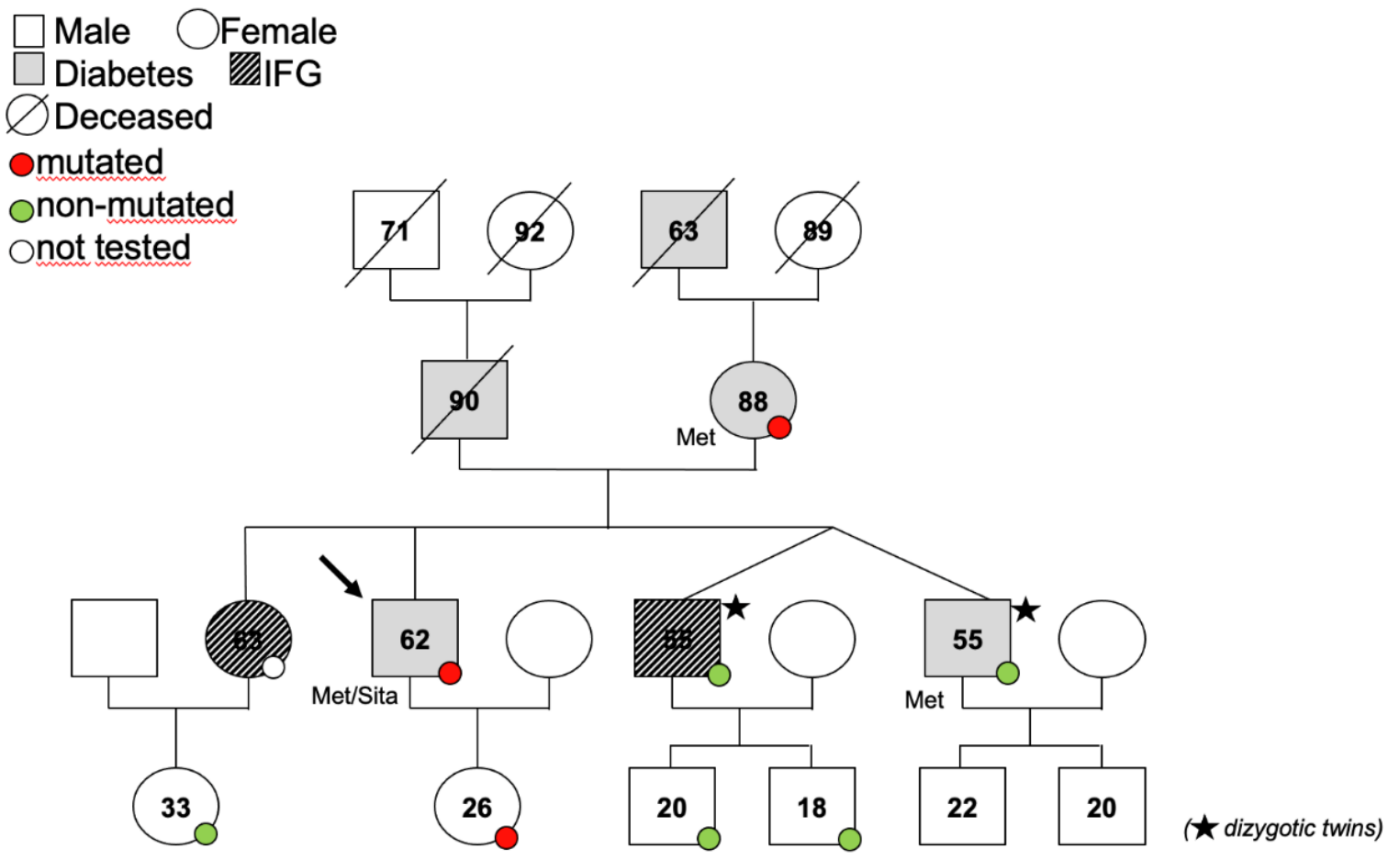
Pedigree of family of case 1. The black arrow indicates the proband. For living members, the number inside the symbol indicates respective age at the time when the genetic test was performed. For the deceased (slashed symbols), the number indicates the age at the time of death. At the time of genetic testing, maternal grandmother could not be tested because already deceased, and proband’s daughter had normal weight, had never been diagnosed with diabetes, and had never undergone a pregnancy. Concomitant antidiabetic treatment is shown underneath respective symbol. Met = metformin; sita = sitagliptin

A 62-years old male patient of European ancestry was referred for a history of diabetes diagnosed at the age of 53 after having had IFG for 13 years. Diabetes affected both his parents, the maternal grandmother and a brother since the age of 50; two other brothers had dysglycemia. There was no family history for CVD or autoimmune diseases. The patient had no detectable islet-cell autoantibodies and C-peptide levels were normal. BMI was 22.2 kg/m^2^. He conducted a healthy lifestyle and medical history was otherwise unremarkable. He was receiving metformin 1 g BID and was not taking any other medication. When HbA1c raised to 7% under metformin monotherapy he was switched to sitagliptin/metformin 50/1000 mg BID. In view of the family history and the lack of other typical features of T2D, genetic testing was performed for monogenic forms of diabetes. A new heterozygous missense mutation was detected in the *ABCC8* gene (p.Ile1075Thr, c.3224T > C), resulting in an amino acid change at a conserved residue across species. The p.Ile1075Thr variant has been reported in heterozygosity in 12/556,006 non-Finnish European individuals in the gnomAD v4 database, and listed in ClinVar 5 as of likely benign/uncertain significance. While the variant in silico analysis did not univocally predict a deleterious effect of the mutation on the protein function (Polyphen2 0.062; SIFT 0.061; MutationTaster 1.1; CADD PHRED 24.1; MutationAssessor 1.73, REVEL score 0.707), the clinical context may suggest a role of this variant in proband’s diabetes development. The mutation was confirmed in the affected mother and in the not-yet affected 26-years old daughter (Fig. 2). Under sitagliptin/metformin, the patient’s glycemic control was good (HbA1c = 6.3%) thereby not requiring a trial with sulfonylurea.

### Case #2

**Fig. 3:**
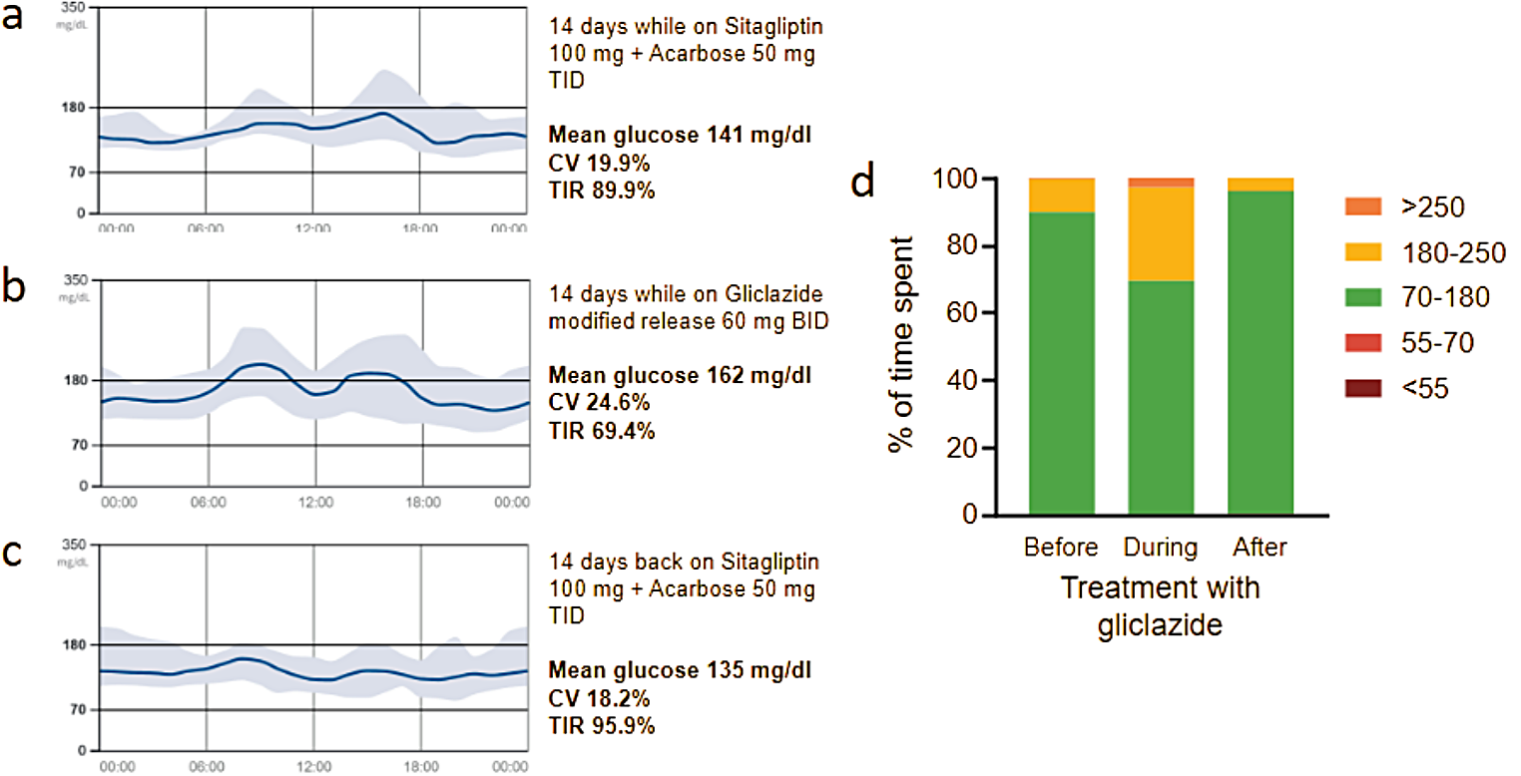
Trial of sulphonylurea treatment in case 2. (**a**) Ambulatory glucose profile (AGP) of 14 days during prior treatment. (**b**) AGP during 14 days of treatment with gliclazide titrated up to 120 mg daily. (**c**) AGP during 14 days while the patient was back to the prior therapy. (**d**) Time in range during the three periods

The proband was a 38-years old man of European ancestry with diabetes since the age of 18. There was no family history of diabetes in first or second-degree relatives. At time of diagnosis, islet-cell autoantibodies were negative and a glucagon test revealed beta-cell dysfunction (baseline 0.3 ug/l ◊ post-glucagone 0.5 ug/l). Therapy with metformin was initiated at the age of 27 years because of HbA1c elevation above 7% (53 mmol/mol). During metformin monotherapy, the patient experienced a few episodes of symptomatic hypoglycaemia with confirmed glucose values of about 50 mg/dl (2.8 mmol/l). Meanwhile, beta-cell secretion appeared to have improved (fasting 1.2 ug/l ◊ post-breakfast 2.9 ug/l). Re-testing of islet cell autoantibodies 20 years after diagnosis were still negative (ICA, GADA, IA2A, anti-Znt8). Due to a rise in HbA1c up to 7.5% (58 mmol/mol), therapy with sitagliptin and acarbose were added and metformin was withdrawn because of recurrent episodes of hypoglycaemia. While under sitagliptin 100 mg and acarbose 50 mg TID, HbA1c fell to 6.5% (48 mmol/mol) and no hypoglycaemia were reported after stopping metformin. Body weight was 68 kg and BMI was 21.7 kg/m^2^. There were no risk factors for T2DM (hypertension, dyslipidemia, cardiovascular disease). The Exeter calculator provided a 75.5% probability of MODY. Whole exome sequencing for MODY genes revealed the c.1642 A > C variant in the *ABCC8* gene, resulting in the amino-acid substitution Thr548Pro, located in a conserved trasmembrane protein portion of TDM1 (phyloP score Vertebrate 2.17/6.42; Primate 0.52/0.65. PhastCons 1.00/1.00). Bioinformatic analysis did not strongly predict pathogenic changes in protein structure or function (Polyphen 0.157/1.00; SIFT 0.04/0.00; MutationTaster 0.998/1.000; CADD PHRED 16/20; MutationAssessor 1.5/5.0; REVEL score 0.596). The mother and father did not carry the proband’s genetic variant. The variant was previously reported only in a cohort of individuals with common T2DM [58], but never in ABCC8-related diseases, including ABCC8-MODY, and is not listed in the gnomAD database. While such variant was considered of uncertain pathogenic significance, the patient’s clinical history led to a diagnosis of ABCC8-MODY. Consequently, the therapy was switched from the sitagliptin/acarbose combination to gliclazide modified release, uptitrated to 60 mg BID, while the patient was wearing an intermittently scanned CGM. After 1 month, glucose control had deteriorated with prevalent post-prandial hyperglycaemia. Therapy with sitagliptin/acarbose was then reinstalled, which allowed a good and stable glycaemic control (Fig. 3).

### Case #3

The proband was a 34-year-old woman of European ancestry with a recent diagnosis of diabetes upon OGTT (fasting 115 mg/dl ◊ 2hr post-load 203 mg/dl). Fasting C-peptide was 1.2 ug/l and HbA1c was of 5.1% (33 mmol/mol). She had a history of Hashimoto’s thyroiditis, but islet-cell autoantibodies scored negative (ICA, GADA, IA2A, anti-Znt8). Her body mass index was 19 kg/m^2^. She had no family history of diabetes in first-degree relatives and no personal risk factors for T2DM. HOMA-B was 34.2, which is considerably lower than what observed in normal glucose tolerance individuals of European ancestry (range 117.9–144.0) [71]. In addition, her insulinogenic index calculated during OGTT was 0.19, again considerably lower than the normal range of 1.06 ± 1.10 (mean ± SD) reported before [72]. These data point to a clear insulin secretory defect. According to the Exeter calculator, the probability of the patient having MODY was 75.5%. Whole exome sequencing for MODY genes revealed the variant c.3682G > A in the *ABCC8* gene, resulting in the amino-acid substitution Glu1228Lys, located in a moderately conserved position (phyloP Vertebrate 2.12/6.42; Primate 0.56/0.65; PhastCons 0.97/1.00) in a transmembrane portion of TMD2. Such variant is reported in 17/589,998 and 38/806,991 European (non-Finnish) individuals and individuals across all genetic Ancestry Groups respectively in the gnomAD v4 database. The bioinformatics analysis did not suggest a potential pathogenic effect on protein structure or function (Polyphen2 0.006/1.00; SIFT 0.068/0.00; MutationTaster 1.00/1.00; CADD PHRED 13/20, MutationAssessor 1.17/5.00; REVEL score 0.289). Thus, despite the absence of any reasonable alternative diagnosis, ABCC8-MODY could not be univocally confirmed. The patient HbA1c remained within normal value and one year after diagnosis, she is not being treated pharmacologically.

## Conclusions

None of the three newly-described cases satisfy the classical diagnosis of ABCC8-MODY (Table 2). Features against a diagnosis of ABCC8-MODY are: (i) onset in adulthood; (ii) uncertain pathogenicity or likely benign nature of the ABCC8 mutations; (iii) good glycemic control without sulphonylurea or insulin. Yet, the patients do not even appear to have classical T2D because of early onset (except case 1), normal weight, lack of other cardiovascular risk factors and features of insulin resistance. With BMI ranging from 19 to 22 kg/m^2^, it is hardly arguable that these patients have excess adipose tissue even considering the personal fat threshold hypothesis [73] (but with the caveat of considering ectopic fat sites). Therefore, diabetes in these patients should be most likely attributed to primary beta-cell dysfunction. Thus, in the absence of other possible causes of diabetes, it seems reasonable to hypothesize a role of the identified ABCC8 gene variants in such insulin secretory dysfunction. As discussed above, the functional consequences of *ABCC8* mutations are extremely heterogeneous, ranging from neonatal hypoglycemia to early onset and late onset diabetes, with quite limited genotype-phenotype correlation. Therefore, it could be arguable that, in case of insufficient glycemic control, an attempt to treatment with sulphonylurea should be performed in individuals with a clinical history suggestive for MODY. It should be noted that some *ABCC8* variants predict good response to incretin-based therapies, which may explain why two out of two patients who needed treatment were well controlled while on sitagliptin. Of particular interest is the history of hypoglycemia in case 2. As hypoglycemia due to metformin is very rare, it is tempting to speculate that the identified *ABCC8* variant may be involved.

In summary, we describe three controversial clinical cases, in whom the identification of novel *ABCC8* variants in heterogeneous clinical settings led to a suspect of ABCC8-MODY, that could not be unequivocally confirmed by the pathogenicity analysis of the detected variants.

What emerges from the data discussed in this review is the unsolved elusiveness of genotype-phenotype interactions in ABCC8-MODY. Although *ABCC8* is an established MODY-related gene, the functional effects of many of its variants remain unclear, and this knowledge gap contributes to ABCC8-MODY misdiagnosis. Thus, further studies are warranted to clarify the role of detected *ABCC8* mutations in the pathogenesis of the disease and how they influence clinical features and the response to therapy. When translated into clinical practice, this information will be of great significance for an early diagnosis, individualized selection of appropriate treatment, and amelioration of prognosis.

## Data Availability

No datasets were generated or analysed during the current study.
